# An Unusual Encounter: Aeromonas veronii Infection in a Case of Ulcerative Colitis

**DOI:** 10.7759/cureus.52010

**Published:** 2024-01-10

**Authors:** Reshmi Adupa, Harika Dadigiri, Toufiq Imtiaz

**Affiliations:** 1 Internal Medicine, New York Medical College Program at St. Mary’s General Hospital and St. Clare’s Health, Denville, USA; 2 Internal Medicine, Sri Venkateswara Medical College, Tirupati, IND

**Keywords:** bloody stool, antibiotic therapy, aeromonas infection, ulcerative colitis, aeromonas veronii

## Abstract

*Aeromonas veronii*-associated ulcerative colitis flare is sparsely reported in the literature but is a treatable condition with antibiotics including trimethoprim/sulfamethoxazole (TMP-SMX), fluoroquinolones, and second/third-generation cephalosporins. We report a case of a patient with long-standing ulcerative colitis (UC), who presented with bloody stools, fatigue, and oliguria that did not respond to standard regimen including steroids. The lab finding was significant for leukocytosis and anemia. *A. veronii* was cultured in the stool specimens. The patient was started on ciprofloxacin with marked improvement of symptoms on the second day of initiation of the antibiotic. Although rare, a possible Aeromonas infection should be suspected in patients presenting with a flare of ulcerative colitis. A prompt initiation of treatment can provide rapid improvement in clinical status of these patients.

## Introduction

Aeromonas veronii is a bacteria with a rod-shaped and motile structure which is classified as Gram-negative. It mainly thrives in freshwater environments such as groundwater, lakes, and rivers. Among the diseases caused by Aeromonas species in humans, the most prevalent ones are gastroenteritis, soft-tissue infections, and bacteremia. The human gastroenteritis associated with Aeromonas species is primarily caused by three specific species as follows: A. veronii, Aeromonas caviae, and Aeromonas hydrophila, with A. veronii being the species that is most frequently isolated [1]. However, there has been conflicting information regarding its role as a gastrointestinal pathogen, as it is not uncommon to find it in the stool of asymptomatic individuals. The most frequent manifestation of Aeromonas infection is diarrhea, which is typically sudden and resolved on its own. However, there have also been reported cases of bloody diarrhea and abdominal pain, as well as chronic and less severe diarrhea [2]. Different types of Aeromonas species possess various virulence factors, such as cytotoxic and cytotonic toxins, proteases, hemolysins, lipases, adhesins, agglutinins, pili, enterotoxins, and several other enzymes. However, there is still limited knowledge regarding the prevalence of these virulence factors in Aeromonas and their exact role in the pathogenesis of human enterocolitis. Additionally, Aeromonas has been suggested as a potential trigger for flares in inflammatory bowel disease (IBD) and the development of de novo colitis in individuals with no previous history of IBD [3].

## Case presentation

A 67-year-old female patient was admitted to the hospital with an increased frequency of bloody diarrhea, fatigue, and decreased urine output. Patient’s past medical history included hypothyroidism and ulcerative colitis which was diagnosed a year ago. The patient had a history of intermittent bloody stools last year, however, this episode was associated with >10 bloody bowel movements per day. Over the past year, she had remained on mesalamine 1 g suppositories and steroid foam. Her last colonoscopy a month before her admission was consistent with colitis, which extended from distal sigmoid to rectum. In addition, she had traveled to Jamaica for a week, a month before her admission. She denied fever, chills, nausea, vomiting, abdominal pain, dysuria, significant weight loss, and similar complaints in her family. Physical examination showed conjunctival pallor, dry mucous membrane, no abdominal tenderness, distention, guarding, or rigidity with normal bowel sounds. Vital signs are heart rate of 78-82 beats per minute, blood pressure was 108/68 mmHg, and SpO_2_ of 98% on room air.

The laboratory studies revealed a total leukocyte count (TLC) of 14,400 cells/mm^3^ (normal {n}=5000-10,000 cells/mm^3^) and absolute neutrophil count (ANC) of 13,000 cells/uL (n=2500 cells/uL), hemoglobin 9.4 g/dL (n=11.6-15 g/L), hyponatremia with sodium 127 mmol/L (n=135-145 mmol/L), hypochloremia with chloride 89 mmol/L (n=96-106 mmol/L), hypokalaemia with potassium 2.7 mmol/L (n=3.5-5.0 mmol/L), creatinine 1.2 mg/dL (n=0.57-1.11 mg/dL), and hypoalbuminemia 1.6 g/dL (n=3.4-5.4 g/dL). Serological markers testing was as follows: perinuclear anti-neutrophil cytoplasmic antibodies (pANCA)-positive and negative anti-Saccharomyces cerevisiae antibody (ASCA).

**Figure 1:**
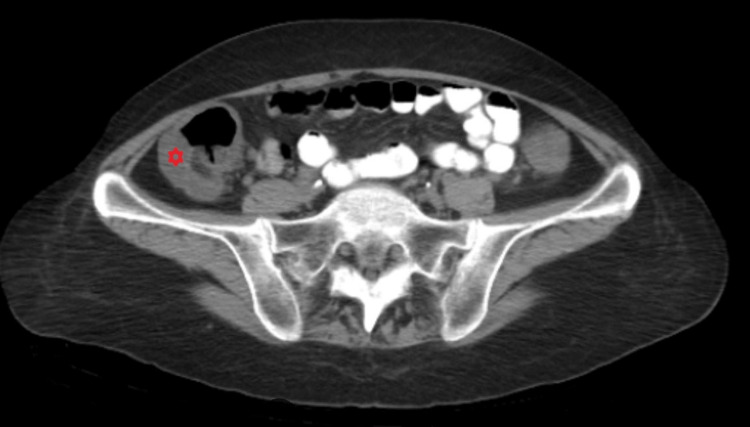
CT scan shows diffuse wall thickening and edema of colon from the right hemicolon to the rectum.

CT scan abdomen findings shown in Figure 1 revealed diffuse wall thickening and edema of the colon from the right hemicolon to the rectum, which was suggestive of known ulcerative colitis. Stool *Clostridium difficile* assay, ova and parasites, Giardia antigen, and GI pathogen panel were negative. Stool cultures were positive for *A. veronii *on day 4 and confirmed the sensitivity to all antibiotics, including fluoroquinolones.

Intravenous steroids and intravenous fluids were initiated on day 1 for ulcerative colitis flare-up. Electrolytes were corrected and nutrition optimized. There was no clinical response by day 4 of admission. On day 4, she was started on ciprofloxacin 500 mg PO twice daily for three days for *A. veronii* infection. Her diarrhea started to resolve after the initiation of ciprofloxacin therapy, and on day 6, her bowel movements came down to 3-4 per day. The patient was discharged on day 8 on PO prednisone and the biological agent ustekinumab was initiated by her gastroenterologist with further outpatient follow-up.

Within two days after initiation of PO ciprofloxacin, the patient's bowel movement frequency came down to 2-3 episodes per day. The patient felt symptomatically better and was discharged home to continue the best supportive care at home, with further follow-up scheduled with a gastroenterologist.

## Discussion

Despite being initially reported in 1891, it was only in 1954 that the potential of Aeromonas as a human pathogen was recognized. These microorganisms were identified in clinical samples obtained from an immunosuppressed woman who sadly passed away due to severe metastatic myositis [4]. The role of Aeromonas in causing human enterocolitis is still subject to debate, as is its association with the development of inflammatory bowel disease (IBD) or triggering flare-ups. The prevalence of Aeromonas gastrointestinal infection worldwide varies from 2% to 88%, and its carriage rate in healthy individuals ranges from 1% to 45%. The rates of stool isolation differ depending on factors such as geographical location, dietary habits, and isolation methods. In developed countries, the prevalence of infection ranges from 0.8% to 7.4%, and the carriage rate ranges from 0% to 4% [2]. Although it remains unclear what exactly causes inflammatory bowel disease (IBD), there are numerous factors at play within its network of pathogenic mechanisms, including environmental influences, genetic susceptibility, dysregulated immune responses, and microbiological factors. In relation to the latter, both changes in the intestinal microbiota and infections caused by external agents may contribute to the onset and exacerbation of IBD.

Dysbiosis can also result from commensal flora that, while normal in terms of species, possess more subtle virulence factors like enteroadherence or a lack of diversity in the fecal microbiome [2]. These organisms can be best identified by utilizing culture methods, performing a Gram stain, and conducting biochemical tests. A positive oxidase reaction, growth in nutrient broth without the presence of NaCl, lack of growth in nutrient broth containing 6% NaCl, inability to thrive on thiosulfate citrate bile sucrose agar, and resistance to the vibriostatic compound 0/129 can aid in distinguishing Aeromonadaceae from the genera Vibrio and Plesiomonas [5]. The clinical presentation in Aeromonas infection of the intestinal tract typically manifests as enteritis, characterized by a short bout of acute diarrhea lasting approximately two weeks. This is frequently accompanied by abdominal discomfort and fever, and occasionally accompanied by vomiting. In certain cases of Aeromonas-associated diarrhea, the clinical features may be suggestive of ulcerative colitis, with persistent passage of blood and mucus in the stool and the presence of proctitis on sigmoidoscopy [6]. Aeromonas exhibits universal resistance to the narrow-spectrum penicillin antibiotics, such as penicillin, ampicillin, carbenicillin, and ticarcillin. However, they are susceptible to piperacillin, second- and third-generation cephalosporins, and carbapenems. A majority of Aeromonas species show susceptibility to aminoglycosides, tetracycline, chloramphenicol, trimethoprim-sulfamethoxazole, quinolones, and monobactams [5]. To prevent these infections, it is important to maintain proper sanitary conditions. This includes practicing good hand hygiene, ensuring efficient sewage disposal, and following hygienic food preparation practices. Additionally, cooking food thoroughly can help minimize the transmission of the infection. It is also crucial to properly dispose of any diseased animals and treat water appropriately to prevent the spread of Aeromonas [5].

## Conclusions

In conclusion, this study highlights an uncommon instance of *A. veronii *infection in a patient with ulcerative colitis, presenting an individual and complex clinical situation. This case emphasizes the significance of considering unusual infections in patients with underlying inflammatory bowel diseases. Healthcare professionals should maintain a high level of suspicion and thoroughly evaluate clinical manifestations and laboratory findings, particularly in cases that do not respond to standard therapies.

Early recognition and proper management are vital to achieving successful outcomes in such infrequent infections. Further research is necessary to acquire a deeper understanding of the underlying causes and optimal treatment strategies for *A. veronii *infections in patients with ulcerative colitis.
